# Lignocellulose-derived inhibitors can extend residence of *Clostridium beijerinckii* in active solventogenic state

**DOI:** 10.1186/s40643-025-00871-y

**Published:** 2025-04-09

**Authors:** K. Koppova, L. Burianova, P. Patakova, B. Branska

**Affiliations:** Department of Biotechnology, University of Chemistry and Technology Prague, Technická 5, Prague, 16628 Czech Republic

**Keywords:** Lignocellulose inhibitors, Viability, ABE, Butanol, *Clostridium beijerinckii*, Cytometry, Carboxyfluorescein diacetate

## Abstract

Lignocellulose is a promising renewable resource for producing platform chemicals, such as acetone, butanol, and ethanol, via ABE fermentation by solventogenic clostridia. This study investigates the effects of common lignocellulose derived inhibitory compounds: ferulic acid, coumaric acid, and furfural on *Clostridium beijerinckii*. Dual-staining with propidium iodide and CFDA, combined with flow cytometry, was employed to assess physiological variability. The results showed that phenolic acid-induced stress helped maintain a higher proportion of viable cells during the production phase, enhancing solvent yields and reducing sporulation. At 0.4 g/L, ferulic and coumaric acids did not reduce cell viability; however, coumaric acid exposure led to an acid-crash profile. Conversely, a more robust inoculum exposed to both phenolic acids simultaneously exhibited effects similar to ferulic acid alone, including slower viability decline, reduced growth and sporulation, and improved solvent production. Furfural exposure at 1.5 g/L resulted in immediate viability loss in 20% of the population, though the overall decline accompanied by the highest sporulation rate occurred later than in the control. Additionally, furfural transformation was slower, suppressing butyrate production and reducing solvent production by 13%. This study suggests that delaying cell death mechanism may explain the stimulatory effects of inhibitors, advancing lignocellulose use in the future.

**Supplementary Information:**

The online version contains supplementary material available at 10.1186/s40643-025-00871-y.

## Graphical abstract

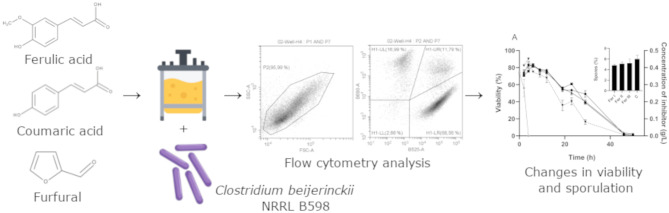

## Introduction

Despite numerous challenges, lignocellulose remains one of the most promising renewable resources for the biotechnological production of platform chemicals. Among the valuable biochemicals derived from this biomass are solvents, such as butanol, acetone, ethanol and/or isopropanol (Sedlar et al. 2021), produced by solventogenic clostridia. However, both the conversion of lignocellulose into substrates suitable for microbial fermentation and the subsequent solvent production processes face many challenges. These obstacles undermine both the economic viability and the ecological sustainability of the overall process. One of the critical issues is the presence of inhibitory compounds generated during the pretreatment of lignocellulose (Baral and Shah 2014). These inhibitors can significantly hinder or even completely block the effective microbial utilization of hydrolysed substrate. Therefore, numerous studies have focused on removing such inhibitors from hydrolysates. While these detoxification strategies often raise economic costs and environmental burdens due to additional processing requirements. Solventogenic clostridia offer a distinct advantage; they not only utilize a wide spectrum of saccharides (Servinsky et al. 2010) but also detoxify various inhibitors through their innate degradation pathways (Ujor and Okonkwo 2022). However, while most inhibitor effects are deleterious to production performance, certain inhibitors have been observed to paradoxically stimulate solvent production under specific conditions (Ezeji et al. 2007; Luo et al. 2019). To this date, no comprehensive study has yet been conducted on the direct physiological effects of inhibitors on solventogenic clostridia. A study by Liu et al. (2018) focused on the changes in the viability of *C. beijerinckii* induced by coumaric acid; however, the assay primarily covered the overall changes in cell growth and metabolic activity. The presumed effects of lignocellulose-derived inhibitors are thus largely based on studies of other organisms and more common transcriptomic studies (Patakova et al. 2022). Among the most notable impacts are direct effects on DNA and its repair mechanism (Ujor et al. 2015; Hadi et al. 1989), disturbance in reducing power levels, an increased need to cope with ROS (Liu et al. 2018; Luo et al. 2021), alterations in transmembrane transport (including both efflux pump activities (Jiménez-Bonilla et al. 2020; Lee et al. 2015) and substrate uptake (Luo et al. 2020), and the activation of a general stress response in cells (Patakova et al. 2022). As a result, reduced growth and cell density, decreased substrate utilization, and overall lowered solvents production have been reported elsewhere (Luo et al. 2021). The unusual biphasic metabolism of clostridia, combined with their sporulation cycle, adds to the complexity of the issue.

The widely accepted features of ABE fermentation include an initial exponential growth phase, during which cells produce acetic and butyric acids, leading to rapid acidification of the growth medium. To alleviate this, the metabolism shifts to solvent production, where acids are reutilized and pH increases. Nevertheless, these solvents are toxic to the cells, and their accumulation eventually halts growth. Further, during this transition from acidogenesis to solventogenesis, cells begin to form endospores.

Direct utilization of non-detoxified lignocellulose hydrolysates or the artificial addition of inhibitory compounds, shifts the production characteristics towards acid formation and reduced solvent production. This can lead to limiting situations, often known as acid-­crash (Baral and Shah 2014; Liu et al. 2015a, 2024), when no pH rise occurs due to the lack of acid reutilization. As shown in numerous available studies, the cell response to the presence of inhibitors is very complex. Their addition affects the expression of hundreds of genes (Patakova et al. 2022), and various strain transformation can lead to improved tolerance (Jiménez-Bonilla et al. 2020, 2024; Lee et al. 2015). Another approach could involve designing a biotechnological process that alleviates the negative effects of inhibitors (Survase et al. 2021; Branska et al. 2024).

Although less frequent, some studies suggest that inhibitors could also have a positive effect, which presents an opportunity to improve the performance of the lignocellulose utilization process (Ezeji et al. 2007; Luo et al. 2019). The stimulation might be attributed to the modulation of regulatory networks that control the switch between two metabolic phases, or it may be caused by better cells adaptation to the subsequent stress of solvents (Liu et al. 2019). Nevertheless, these explanations remain hypotheses rather than direct evidence. Further research is necessary to fully understand these mechanisms and to harness them for biotechnological applications and optimizing lignocellulose utilization. One such tool that enables the direct exploration of physiological changes in culture over time is flow cytometry. Methods to monitor physiology, viability, and sporulation have previously been established to reveal culture dynamics during the life cycle of solventogenic clostridia (Linhová et al. 2012; Branska et al. 2018; González-Peñas et al. 2015).

This study investigates the effects of common inhibitors: furfural, an aldehyde byproduct from the acidic hydrolysis of lignocellulosic biomass, and coumaric and ferulic acids, phenolics prevalent in hydrolysates of annual grasses. Primarily, changes in viability and sporulation rates in *C. beijerinckii* cultures during ABE fermentation were thoroughly studied to investigate the evolution and dynamics of physiological features following the addition of inhibitors. In our experiments, we have utilized *Clostridium beijerinckii* NRRL B598, a strain known for its phenotypic variability under different cultivation conditions (Branska et al. 2018). This strain thus serves as a suitable model for examining the physiological impacts of lignocellulose-derived inhibitors. Our results reveal that coumaric and ferulic acids, while slightly suppressive to the culture growth, may enhance solvent production, likely by prolonging vegetative phase and reducing sporulation. Conversely, furfural exposure also extends the vegetative phase but significantly reduces overall viability and increases sporulation rates, leading to lower final solvent concentrations.

## Methods

### Strain and growth conditions

*Clostridium beijerinckii* NRRL B598 was maintained in the form of spore suspension in sterile distilled water at 4 °C (Sedlar et al. 2017). Prior to inoculation, the spores were activated by heat shock at 80 °C for 2 min and then transferred to 300 mL of TYA medium (20 g/L glucose, 2 g/L yeast extract, 6 g/L tryptone, 0.5 g/L K_2_HPO_4_, 3 g/L ammonium acetate, 0.3 g/L MgSO_4_.7H_2_O, 0.01 g/L FeSO_4_, pH 6.8). The inoculum was cultivated anaerobically at 37 °C for 24 h (30 h for combined phenolic acid challenge) in an anaerobic chamber.

Cultivation was conducted in 4 parallel 1 L bioreactors (Infors HT), each containing 570 mL of TYA medium without saccharides and 70 mL of sugar solution (250 g/L glucose and 150 g/L xylose). The culture medium was first deoxygenated by flushing with nitrogen, followed by the addition of various concentrations of inhibitory compounds (ferulic acid: 0.4 g/L, coumaric acid: 0.4 g/L, ferulic and coumaric acids: 0.3 + 0.3 g/L and furfural: 1.5 g/L) (Sigma-Aldrich), dissolved in 2 mL of ethanol. The inhibitors were added to three bioreactors, while the fourth bioreactor contained only 2 mL of ethanol as a control. Subsequently, the medium pH was adjusted to 6.3, and 70 mL of inoculum was added to each bioreactor. Cultivation was carried out at 37 °C for 50 h. During the process, pH was monitored online (averaged for 5 min intervals for presentation in the figures), and samples were collected throughout the cultivation. Sampling frequency was increased during the initial phase of the experiment, as this period is typically critical for the entire fermentation process.

### Analysis of culture broth

Samples were centrifuged (5 min, 6000 rpm) and filtered through 0.2 μm microfilter into vials. The detection of produced acids and solvents, along with glucose and xylose consumption, was performer using Agilent Technologies 1200 HPLC with an RID detector. The following conditions were applied for this analysis: IEX H + polymer column (Watrex), 250 × 8 mm, 8 μm; with 5 mM H_2_SO_4_ as mobile phase, flow rate of 1 mL/min, injection volume of 20 µL, and column temperature of 60 °C.

Additionally, the same samples were analyzed for inhibitors using an Agilent Series 1260 Infinite UHPLC system with DAD detector. The analysis was conducted under the following conditions: Zorbax Eclipse Plus Phenyl-hexyl column, 4.6 × 100 mm, 1.8 μm; gradient elution with 0.025% H_3_PO_4_ in demi water (A) and acetonitrile (B) as mobile phases, flow rate of 1 mL/min, injection volume of 5 µL, and column temperature of 25 °C. Detection was performed at 280 nm for furfural and at 320 nm for ferulic acid and coumaric acid.

### Culture growth and production parameters

Cell growth was assessed by measuring the optical density at 600 nm (OD_600_) using a spectrophotometer (Varian Cary 50). The specific growth rate during both the acidogenic (1) and solventogenic (2) phases was determined by analyzing the optical density two hours after inoculation *(OD*_*min*_), the optical density at the metabolic switch (*OD*_*switch*_), and the optical density achieved during mid-solventogenesis at the 20th h (*OD*_*max*_), along with the times at which these occurred (*t*_*max*_, *t*_*switch*_, *t*_*min*_). The specific growth rate (*µ*) was calculated using the following formulas:

1 $$\:\mu\:\:\left({h}^{-1}\right)=\frac{{ln}_{\left({OD}_{switch}\right)}-{ln}_{\left({OD}_{min}\right)}}{{t}_{switch}-{t}_{min}}$$

2 $$\:\mu\:\:\left({h}^{-1}\right)=\frac{{ln}_{\left({OD}_{max}\right)}-{ln}_{\left({OD}_{switch}\right)}}{{t}_{max}-{t}_{switch}}$$

The yield (3) and productivity (4) of the product were calculated from its initial (*P*_*0*_) and final (*P*_*fin*_) concentrations, the initial (*S*_*0*_) and final (*S*_*fin*_) substrate concentrations, and the time (*t)* at which no further increase in product levels was observed, using the following formulas:

3 $$\:Yield\:\left(\frac{g}{g}\right)=\frac{{P}_{fin}-{P}_{0}}{{S}_{fin}-{S}_{0}}$$

4 $$\:Productivity\:\left(\frac{g}{L*h}\right)=\frac{{P}_{fin}}{t}$$

### Viability analysis

**Fig. 1:**
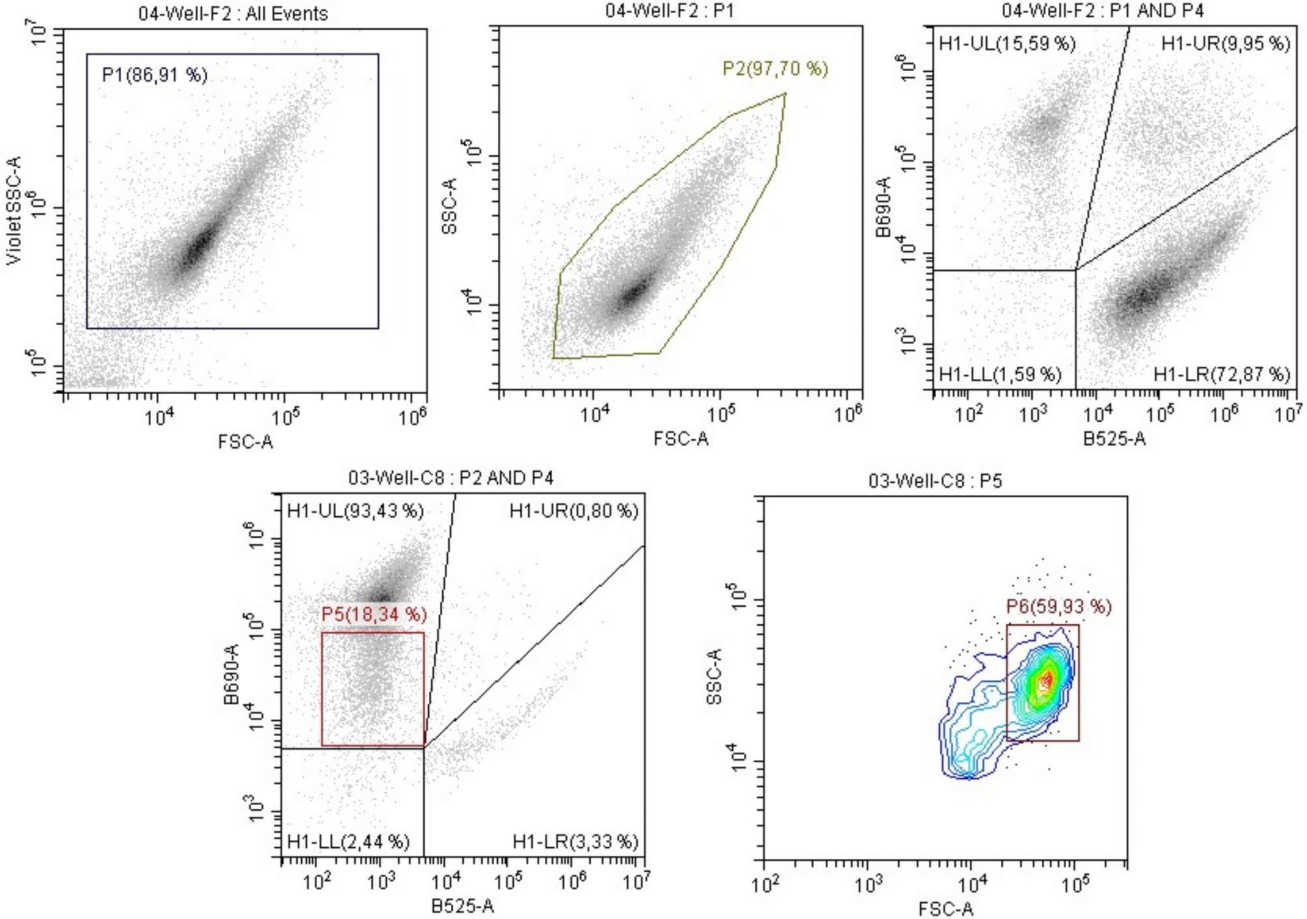
Cytometric data evaluation. Cells were preselected based on the SSC parameter from the violet laser and the FSC parameter from the blue laser (P1). The gate in this group was then adjusted more precisely to capture the majority of the cell population and filter out non-cellular particles (P2). The population thus defined was further constrained by the P4 gate, which is not shown in the figure but defines the time interval in which the particles were analyzed. Selected population was analyzed for fluorescence parameters. The UL quadrant contains propidium iodide-stained cells, the UR propidium iodide and CFDA double-stained cells, and the LR population contains only CFDA-positive, i.e. metabolically active, cells. Two diagrams at the bottom show selection of putative spores (P5) based on their fluorescence pattern and their further analysis for spore specific light scatter parameters (P6)

To assess cell viability, the following protocol was employed. Immediately after sampling from the bioreactor, 10 µL of culture was pipetted into four parallel wells of microplate (starting from OD ≥ 1.0 cells were diluted 5–10 times in saline solution), each containing 190 µL of staining solution (4 µg/mL carboxyfluorescein diacetate (CFDA) and 4 µg/mL propidium iodide (PI) dissolved in saline solution). The samples were mixed thoroughly, incubated in the dark for 7 min, and analyzed using a Cytoflex flow cytometer (Beckman Coulter) equipped with violet (405 nm) and blue (488 nm) lasers. Cells were differentiated from non-cellular particles based on forward scatter (FSC) and side scatter (SSC) parameters (Fig. 1, gates P1 + P2). Fluorescence emission was measured using blue laser at 525 nm and 690 nm to identify CFDA and PI-positive cells, respectively. Active viable cells were defined as the population emitting a fluorescence signal exclusively in the CFDA channel (Fig. 1, gate H1-LR). Compensation was applied, when necessary, with a 3-­5% reduction in red fluorescence based on green emission. The population showing none of the fluorescence (Fig. 1, gate H1-LL) was excluded from the analysis and the representation of cells in the other quadrants was calculated to 100%. Spores were identified as described by Branska et al. (Branska et al. 2018). Briefly, particles displaying FSC and SSC properties consistent with cell-like particles and exhibiting reduced red fluorescence (due to the limited permeability of the spore envelope) were gated (Fig. 1, gate P5). These particles were further analysed for FSC and SSC characteristics to distinguish a uniform spore population from pleomorphic vegetative forms and cell debris (Fig. 1, gate P6). Sporulation rate was calculated as a ratio to all particles identified as cells.

### Data analysis

A one-way analysis of variance (ANOVA) with Dunnett’s multiple comparison test was applied to evaluate differences in production parameters and specific growth rates between each inhibitor­treated culture and the control culture, with a significance level of α = 0.05. For pairwise comparisons among treatments, a one-way ANOVA with Tukey’s multiple comparison test was used. Statistical analyses were performed using GraphPad Prism software. Significance levels were defined as follows: *p < 0.05, **p < 0.01, and ***p < 0.001.

## Results

The carbohydrate concentration and ratio were designed to simulate the potential composition of wheat straw hydrolysate, comprising 25 g/L glucose and 15 g/L xylose. As previously demonstrated (Branska et al. 2018; Branska et al. 2021) (and further corroborated by the results presented here), the physiological state of the culture changes dynamically over time due to production characteristics and the sporulation cycle. Despite strict adherence to established protocols, it is impossible to consistently replicate the same physiological state of the inoculum, as these states can shift within minutes. Therefore, control experiments were consistently conducted in one parallel bioreactor with the biological triplicate for inhibitory challenges. Curves for control are displayed as dashed lines without error bars, in comparison to the mean values of the three challenged biological replicates, which include standard deviations.

This approach differs for cytometry data, where the percentages of live cells are shown for each bioreactor individually to emphasize biological variability or similarity. Here, error bars represent the variability of the assay based on four technical replicates.

The assessment of cell viability was conducted using a combination of propidium iodide (PI) and carboxyfluorescein diacetate (CFDA) staining, which allows for differentiation of cell populations into at least two groups based on distinct staining patterns. One group comprises cells with compromised cytoplasmic membrane integrity, allowing PI uptake. In viable cells, PI is efficiently excluded from the intracellular environment. Once inside compromised cells, PI binds to nucleic acids, causing a several-fold increase in red fluorescence intensity.

In contrast, CFDA is a lipophilic, neutral compound that diffuses freely across intact cytoplasmic membranes into the cytosol. There, intracellular esterase activity cleaves the non­fluorescent CFDA, producing a positively charged green fluorescent product, carboxyfluorescein, which is retained within the cell. Cells with compromised membranes but residual enzymatic activity may exhibit staining with both fluorescent probes. Similarly, sporulating cells with compromised parent cell membranes but immature spores can also display dual staining. Only cells with a clear positive response to CFDA staining were selected for viable population assessment, as these cells are assumed to be the actively producing population exhibiting the highest metabolic activity.

### Ferulic acid challenge

**Fig. 2:**
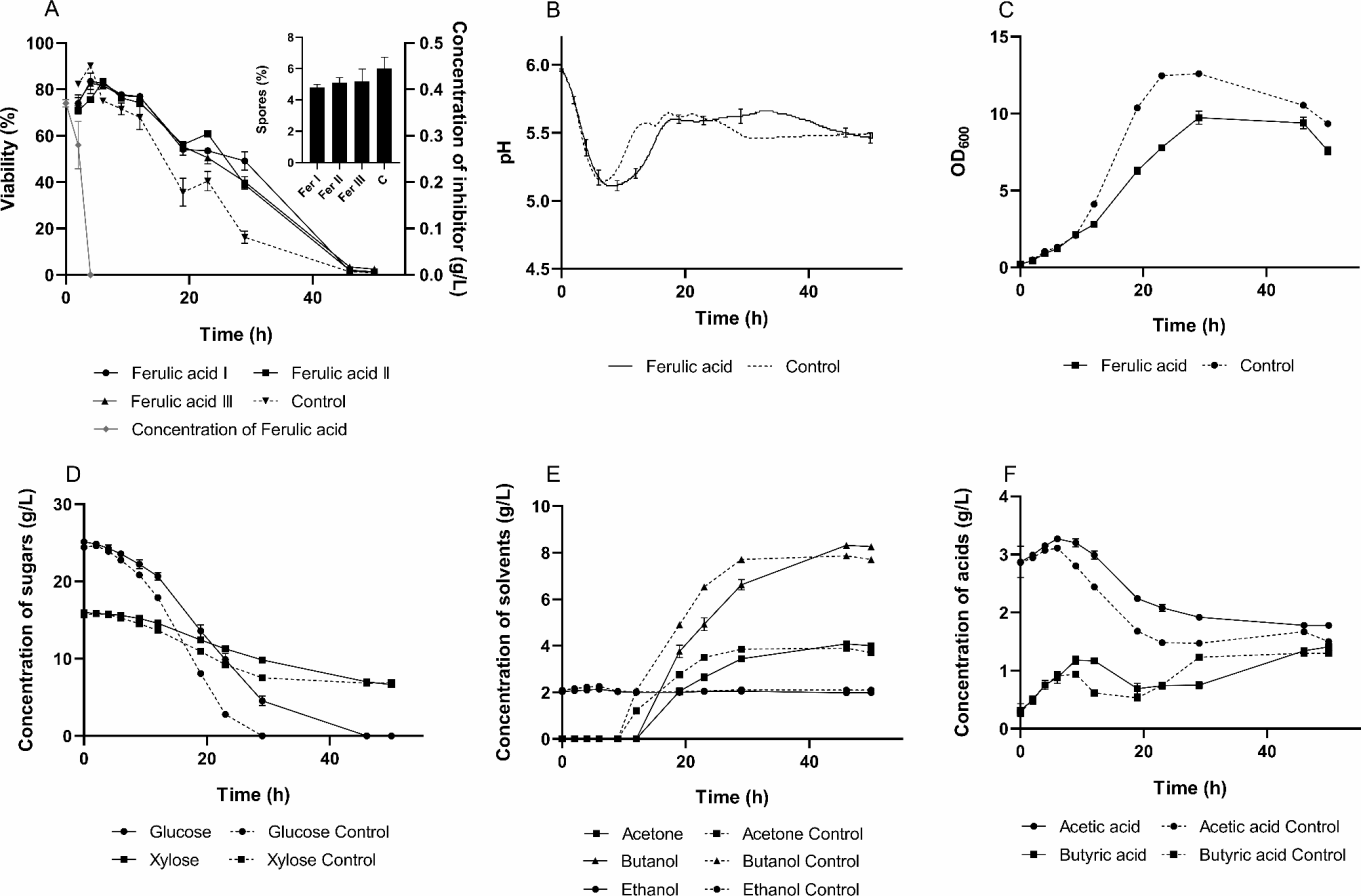
Fermentation characteristics of *C. beijerinckii* NRRL B598 under ferulic acid challenge (0.37 ± 0.01 g/L). (**A**) cell viability with sporulation rate; (**B**) pH during cultivation; (**C**) optical density during cultivation; (**D-E**) concentration of sugars, solvents and acids, respectively, during cultivation. Cultivation with the inhibitor was conducted in biological triplicate. Viability and sporulation rates were analyzed in four technical replicates. The results are presented as the mean of the respective values, accompanied by their standard deviations. Statistical significance for the sporulation rate was indicated as follows: *p < 0.05, **p < 0.01, and ***p < 0.001

The first experiment involved a ferulic acid challenge, with the results summarized in Fig. 2. An addition of 0.4 g/L of ferulic acid was made to the bioreactor, resulting in an initial concentration of 0.37 ± 0.01 g/L, as determined by liquid chromatography. This concentration rapidly decreased to zero within 4 h. During this brief period, the viability, growth curve, and pH profile showed no significant impact from the inhibitor.

Saccharide and metabolite analysis indicated that glucose utilization and acetic acid production were already slightly slowed at this stage. However, these differences were negligible, with more pronounced effects emerging only after complete transformation of ferulic acid. During the metabolic switch that occurred in the stressed culture 1 h later than in the control culture, higher concentrations of both acetic and butyric acids were observed in the medium, along with a pH difference of only 0.03.

Viability assessment revealed high numbers of active cells in both cultures, although the control culture exhibited a slightly lower cell count that began to decline steeply after the first pH maximum post-switch. A similar decline was noted in the stressed culture during the same interval, but its percentage of viable cells remained at least 20% higher throughout the production phase.

Interestingly, despite the higher proportion of viable cells, optical density (OD) measurements indicated slower growth and a lower overall cell density in the stressed culture. This aligns with the slower utilization of glucose and xylose, which eventually reached similar levels in both the challenged and control cultures. Nonetheless, the butanol concentration was 6.4% higher in the stressed culture. In contrast, the sporulation rate in the challenged culture was 17% lower than in the control. These findings suggest that exposure to ferulic acid allowed the culture to maintain a higher proportion of viable cells, which grew more slowly and produced less biomass and spores.

### Coumaric acid challenge

The experiment with coumaric acid was designed similarly to the ferulic acid trial. An amount equivalent to 0.4 g/L of coumaric acid was introduced into the bioreactors, with liquid chromatography determining an initial concentration of 0.28 ± 0.03 g/L. This concentration rapidly decreased to zero, consistent with the degradation observed for ferulic acid. The discrepancy between the added amount and the measured concentration may be due to the limited solubility of coumaric acid in an aqueous environment, potentially resulting in incomplete dissolution at the time of sampling.

**Fig. 3:**
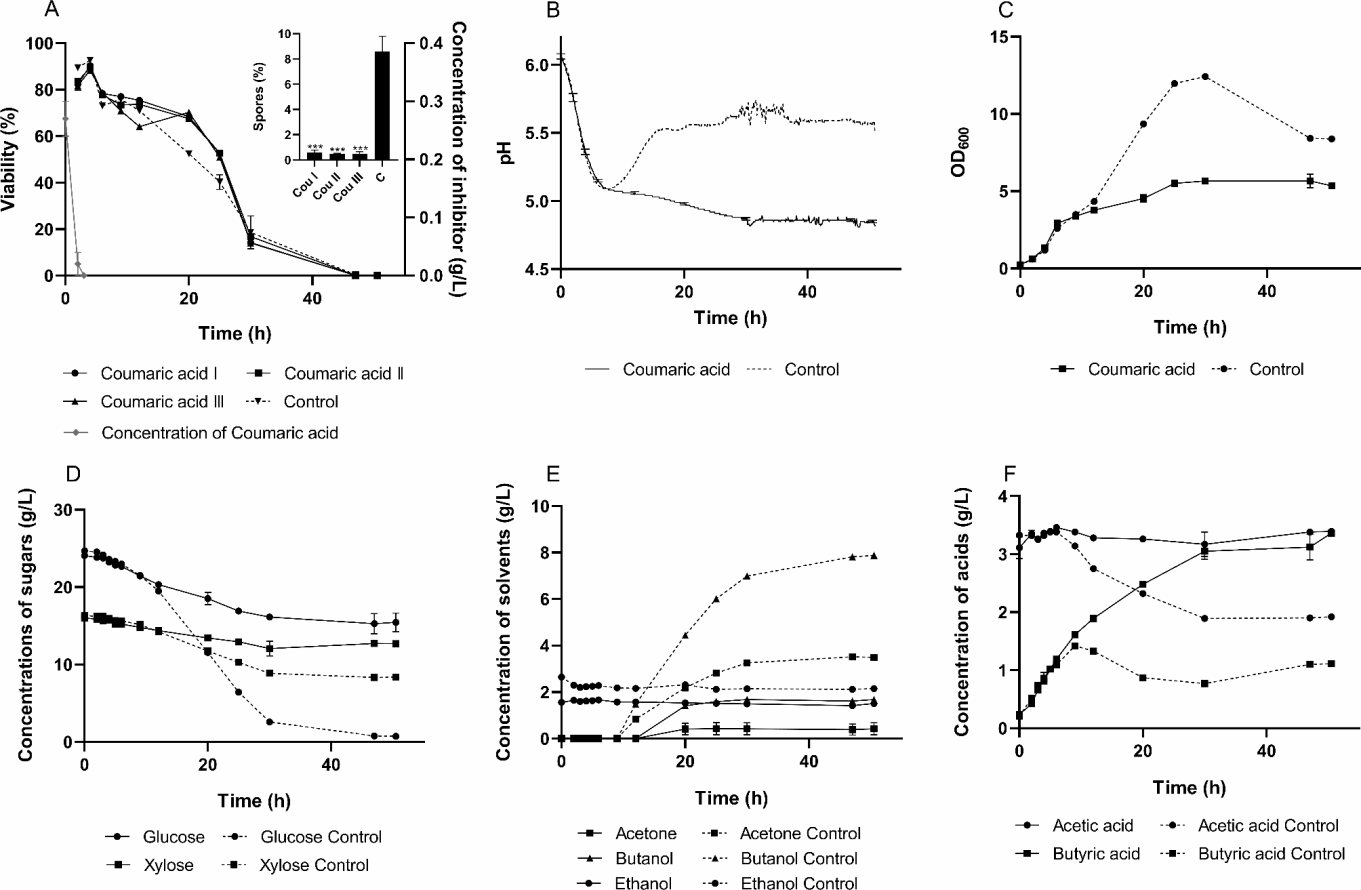
Fermentation characteristics of *C. beijerinckii* NRRL B598 under coumaric acid challenge (0.28 ± 0.03 g/L). (**A**) cell viability with sporulation rate; (**B**) pH during cultivation; (**C**) optical density during cultivation; (**D-E**) concentration of sugars, solvents and acids, respectively, during cultivation. Cultivation with the inhibitor was conducted in biological triplicate. Viability and sporulation rates were analyzed in four technical replicates. The results are presented as the mean of the respective values, accompanied by their standard deviations. Statistical significance for the sporulation rate was indicated as follows: *p < 0.05, **p < 0.01, and ***p < 0.001

During the coumaric acid degradation phase, no notable differences were observed between the challenged and control cultures (Fig. 3). Even after the complete depletion of coumaric acid, data trends across all bioreactors remained consistent until the metabolic transition point. None of the three cultures exposed to coumaric acid transitioned into the acid reutilization mode, and solvent production was delayed, ultimately resulting in an acid-crash in these cultures.

As expected, this fermentation profile was characterized by a zero-sporulation rate, low cell density, poor saccharide utilization, and negligible solvent production. In the initial phase, the viability course resembled that of the ferulic acid experiment. There was even observable that the coumaric acid-challenged culture maintained a higher proportion of viable cells compared to the control sporulating culture. However, this was short-lived, as viability rapidly declined later in the fermentation process, resulting in cell death.

### Furfural challenge

**Fig. 4:**
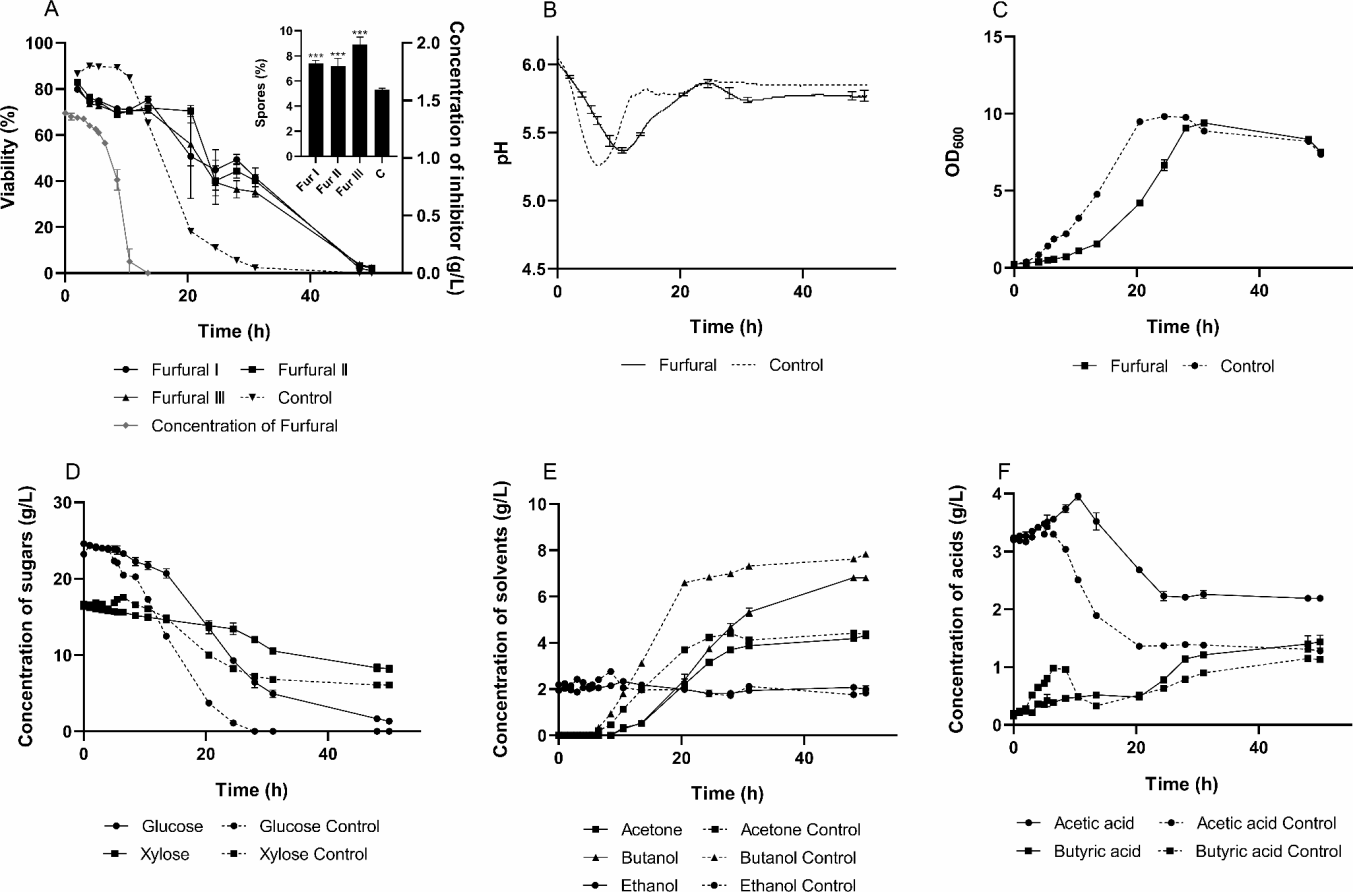
Fermentation characteristics of *C. beijerinckii* NRRL B598 under furfural challenge (0.39 ± 0.02 g/L). (**A**) cell viability with sporulation rate; (**B**) pH during cultivation; (**C**) optical density during cultivation; (**D-E**) concentration of sugars, solvents and acids, respectively, during cultivation. Cultivation with the inhibitor was conducted in biological triplicate. Viability and sporulation rates were analyzed in four technical replicates. The results are presented as the mean of the respective values, accompanied by their standard deviations. Statistical significance for the sporulation rate was indicated as follows: *p < 0.05, **p < 0.01, and ***p < 0.001

Distinct physiological features were observed when *C. beijerinckii* was subjected to furfural stress, as summarized in Fig. 4. When cells were transferred to a medium supplemented with 1.5 g/L of furfural (an initial concentration of 1.39 ± 0.02 g/L was determined), viability significantly declined and never fully recovered. The transformation of furfural proceeded much more slowly than that of phenolic acids, showing negligible degradation in the early stages, with a decline only becoming evident from approximately 6.5 h onward. Correspondingly, all processes were delayed, most notably reflected in the slowed pH decrease and reduced OD values, indicating inhibited culture growth.

The metabolite profile revealed limited butyric acid production and a concurrent increase in acetic acid. The metabolic shift occurred at a higher pH (5.37 compared to 5.26 in the control) and lower butyrate concentrations than in the other experiments. This observation aligns with the hypothesis that the culture redirects reduced NADH cofactors to reduce furfural into furfuryl alcohol. During the solventogenic phase, the viability profile showed similar trends to those observed in the ferulic acid experiment, with an improved survival rate. However, due to restricted growth, the overall cell density was significantly lower, resulting in fewer solvent ­producing cells at this stage.

Despite these challenges, the furfural-exposed culture ultimately reached the same optical density as the control but exhibited a higher proportion of spores (7.8% versus 5.3% in the control culture), indicating higher energy demands for sporulation. Final butanol concentration was 13% lower than in the control.

**Fig. 5:**
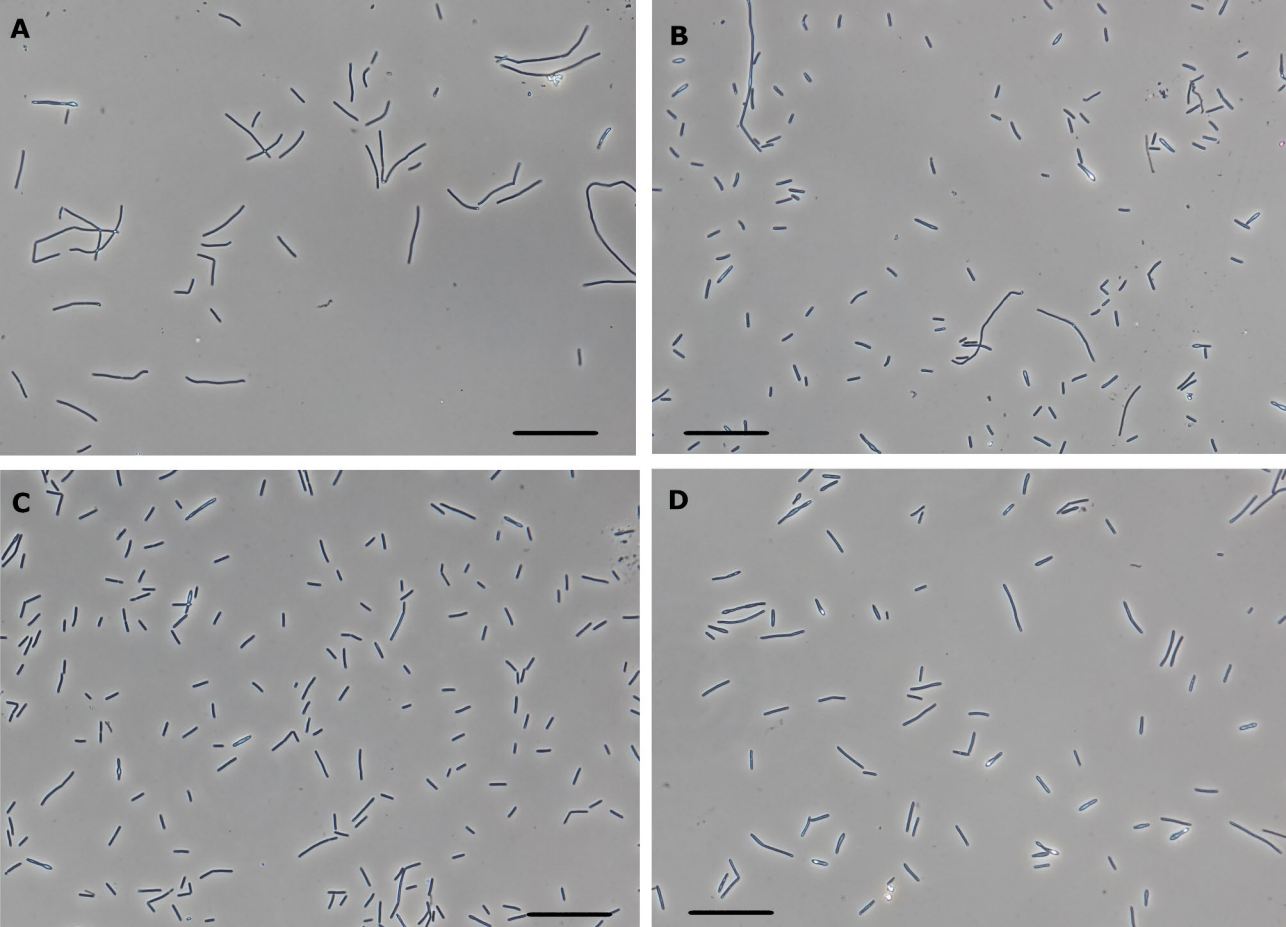
Microphotography of morphological variability of *C. beijerinckii* culture exposed to furfural stress compared to control experiments. Furfural stressed culture at 13.5 h (**A**) and 20.5 h (**B**); control culture at 13.5 h (**C**) and 20.5 h (**D**). The bar represents 40 μm

Cytometric determination of viability at the 20th hour displayed high standard deviation across both biological and technical replicates. Microscopic observations revealed that furfural-stressed cells were highly pleomorphic, undergoing significant morphological transition. Some cells formed long, immotile filaments, that were observed in the stressed culture from the early stages, while others resembled short, motile rods typical of the non-stress phenotype (see Fig. 5 for the comparison of culture morphology). This morphological transition likely varied in degree across each bioreactor at the time of sampling, resulting in an uneven distribution of cell types within the samples. Higher standard deviations were calculated also for technical replicates, making this particular cytometric analysis less reproducible.

### Combined ferulic and coumaric acid challenge using a more resistant inoculum

Earlier findings demonstrated that solventogenic clostridia exhibit varying susceptibility to inhibitors at specific stages of their life cycle (Branska et al. 2024) and are prone to acid-crash, depending on the conditions of the inoculum (Oehlenschläger et al. 2024). Considering these characteristics, we attempted to validate and replicate the results above by conducting a combined inhibitor challenge using ferulic and coumaric acids. To mitigate acid-crash, we inoculated reactors with an older culture already in the early solventogenic phase and introduced both inhibitors simultaneously at concentrations of 0.3 g/L each. The measured concentrations in the medium were 0.27 ± 0.00 g/L for ferulic acid and 0.29 ± 0.00 g/L for coumaric acid. These concentrations were selected to induce a physiological response while avoiding complete inhibition of the culture.

**Fig. 6:**
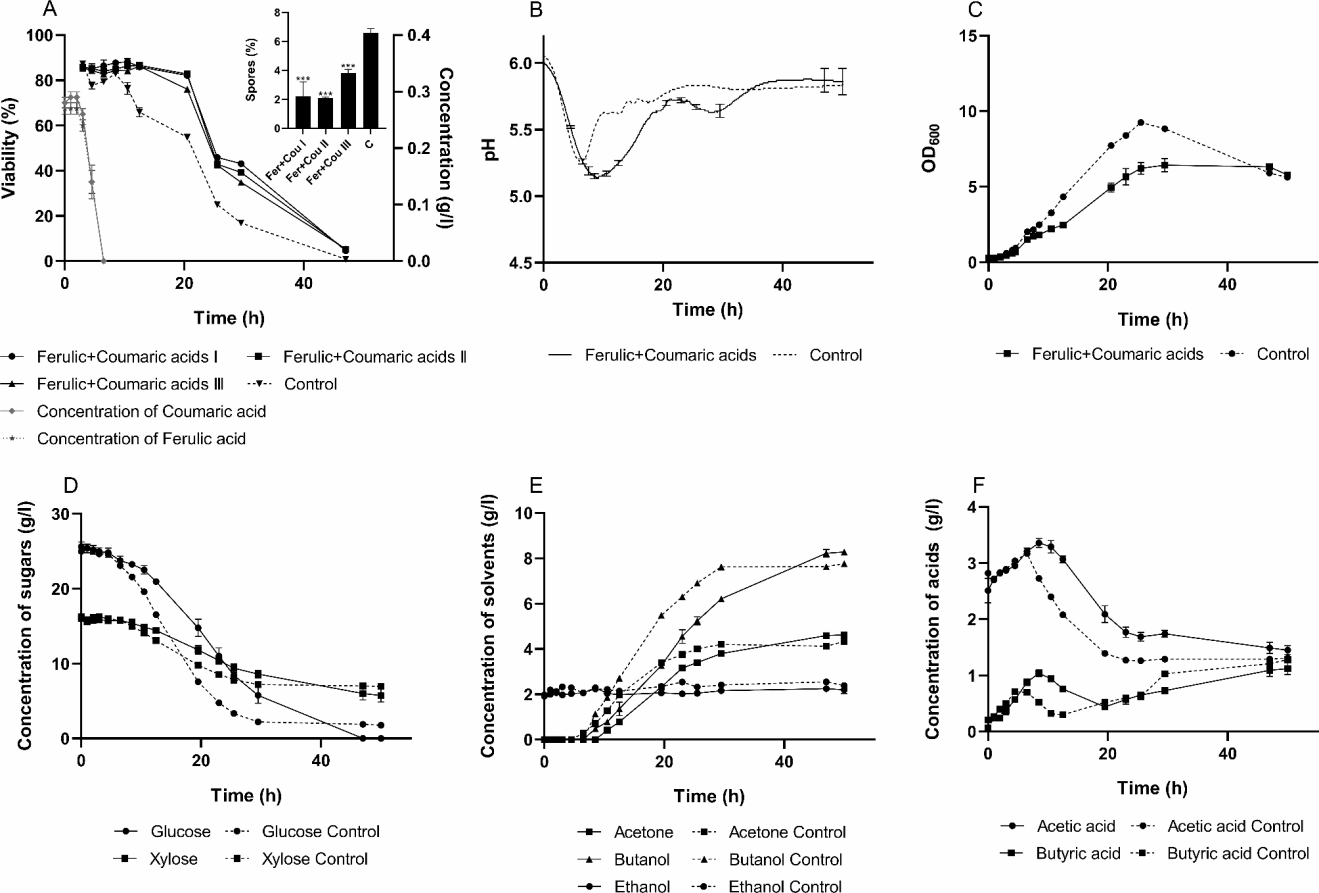
Fermentation characteristics of *C. beijerinckii* NRRL B598 under ferulic and coumaric acids challenge (0.27 ± 0.00 and 0.29 ± 0.00 g/L, respectively). (**A**) cell viability with sporulation rate; (**B**) pH during cultivation; (**C**) optical density during cultivation; (**D-E**) concentration of sugars, solvents and acids, respectively, during cultivation. Cultivation with inhibitors was conducted in biological triplicate. Viability and sporulation rates were analyzed in four technical replicates. The results are presented as the mean of the respective values, accompanied by their standard deviations. Statistical significance for the sporulation rate was indicated as follows: *p < 0.05, **p < 0.01, and ***p < 0.001

The fermentation course was similar to that observed with ferulic acid alone (Fig. 6). The culture maintained a high proportion of viable cells, exceeding 80%, for a longer duration into the later phase of solventogenesis. The transformation of both acids occurred simultaneously without any apparent preference and required more time (6.5 h) compared to the single-acid experiments. The sporulation rate was the lowest among all experiments (excluding the acid-­crash scenario) at 2.7 ± 0.8%, and growth was slower, resulting in a significantly reduced OD.

The metabolic switch was delayed by 3 h and occurred at a lower pH (5.14 compared to 5.26 in the control), which is consistent with the higher concentration of acetate and butyrate at this point. Unlike furfural, this combination of inhibitors did not negatively affect culture viability. Instead, the culture effectively metabolized the acids and followed the standard ABE fermentation pathway. Although the fermentation proceeded at a slightly slower rate than in the control, it yielded a higher butanol concentration in the medium. This improvement is likely attributable to the higher proportion of metabolically active cells, despite the overall lower cell density in the culture.

### Summary of the production parameters

Table 1 provides a summary of the key production characteristics achieved across the experiments. It is important to note that the AB(E) data include only butanol and acetone, as ethanol was added to the medium prior to inoculation to dissolve the inhibitors. Ethanol was chosen because *Clostridium beijerinckii* NRRL B598 produces negligible amounts of ethanol, and the more commonly used solvent, DMSO, interferes with the HPLC separation of solvents.

A comparison of the data in Table 1 reveals statistically significant differences in the final butanol and AB(E) titers, while overall yields of butanol and AB(E) were largely comparable across experiments (except for the acid-crash culture). Butanol productivity was calculated at both 29 and 48 h to account for differences in fermentation time. Specifically, the control cultures reached their final concentrations earlier, resulting in statistically different productivities at these time points. Both metrics indicate that the non-challenged control cultures outperformed the stressed cultures in terms of fermentation rate.

As anticipated, glucose was consumed at a higher rate than xylose in all experiments. The highest xylose utilization was observed in the experiment with combined ferulic and coumaric acid challenges. Conversely, the poorest xylose utilization occurred in the culture challenged with furfural. Nevertheless, Dunnett’s multiple comparison test did not assess any of these values statistically significant, except for the data from the coumaric acid challenge, which led to an acid-crash.

Growth rates were calculated separately for the acidogenic and solventogenic stages. During acidogenesis, the furfural-challenged culture exhibited significantly slower growth with high statistical confidence. In contrast, during the solventogenic stage, this culture recovered and grew at the same rate as the control. The specific growth rates obtained for phenolic acids during solventogenesis were lower than those for the control; however, statistical comparisons revealed that these differences were not statistically significant.

**Table 1:** Comparison of production parameters and specific growth rates in inhibitor-treated and control cultivations

	Ferulic acid 0.37 g/L	Coumaric acid 0.27 g/L	Furfural 1.5 g/L	Ferulic + Coumaric acids 0.28 g/L + 0.27 g/L	Control
Final butanol concentration (g/L)	8.3 ± 0.0***	1.7 ± 0.1***	6.8 ± 0.1***	8.3 ± 0.3**	7.8 ± 0.1
Final AB(E) concentration (g/L)	12.3 ± 0.1*	2.1 ± 0.3***	11.1 ± 0.1	12.9 ± 0.2***	11.8 ± 0.4
Glucose consumption (%)	100.0 ± 0.0	35.9 ± 5.1***	94.6 ± 0.9	100.0 ± 0.0	97.3 ± 2.9
Xylose consumption (%)	56.1 ± 1.9	20.8 ± 0.6***	49.9 ± 3.3	64.2 ± 5.5	56.9 ± 5.3
Glucose + Xylose consumption (%)	83.0 ± 0.9	29.9 ± 3.3***	76.7 ± 1.8	86.3 ± 2.1	81.2 ± 3.1
Butanol yield	0.24 ± 0.00	0.14 ± 0.01***	0.22 ± 0.00*	0.23 ± 0.00	0.23 ± 0.01
AB(E) yield	0.36 ± 0.00	0.17 ± 0.03***	0.35 ± 0.00	0.35 ± 0.01	0.35 ± 0.01
Butanol productivity (g/L.h) at 29 h	0.23 ± 0.01*	0.06 ± 0.00***	0.17 ± 0.01***	0.21 ± 0.00***	0.25 ± 0.01
Butanol productivity (g/L.h) at 48 h	0.18 ± 0.00*	0.03 ± 0.00***	0.14 ± 0.00***	0.17 ± 0.00*	0.16 ± 0.01
AB(E) productivity (g/L.h) at 48 h	0.27 ± 0.00*	0.04 ± 0.01***	0.23 ± 0.00*	0.27 ± 0.01*	0.25 ± 0.01
Specific growth rate - acidogenic phase (h^− 1^)	0.26 ± 0.02	0.39 ± 0.03	0.16 ± 0.02***	0.24 ± 0.02*	0.34 ± 0.05
Specific growth rate - solventogenic phase (h^− 1^)	0.09 ± 0.00	0.03 ± 0.00***	0.13 ± 0.00	0.08 ± 0.00	0.12 ± 0.03

To ensure statistical robustness, each cultivation was replicated three times. The results are presented as the mean value of these three replicates, along with their standard deviation. Statistical significance was calculated by Dunnett’s multiple comparison test and denoted as follows: *p < 0.05, **p < 0.01, and ***p < 0.001. A summary of the statistical differences between treatments can be found in Table S1 in the Supplementary Files.

## Discussion

Efforts to understand the action of lignocellulose-derived inhibitors are motivated by the fact that solventogenic clostridia give very poor production characteristics or do not grow at all on crude hydrolysates, unless some auxiliary intervention is made (Su et al. 2024). In this study, we aim to highlight an unexplored aspect of inhibitor effects, particularly in cases where their role is not strictly negative and may even enhance certain production parameters. Our primary focus is on investigating the ability of the production population to maintain viability. Across all experiments, inhibitor addition led to reduced growth characteristics of both the rate and the overall biomass produced. However, this retardation can paradoxically be beneficial for solventogenic clostridia, as their natural life cycle includes a phase of viability decline (Branska et al. 2018; Kolek et al. 2016) due to spore-forming pathway activation or the effects of accumulating metabolites. This decline is likely linked to the induction of autolytic processes, which are common in clostridia (Liu et al. 2015b) and regulate cell growth, while promoting spore formation by reducing the number of active cells and recycling nutrients. The production of autolysins following the exponential phase has been described for *C. acetobutylicum* ATCC 824. A mutant strain with a deleted gene for the production of one of the autolysins exhibited reduced growth but maintained the same production characteristics (Liu et al. 2015b). Jiménez-Bonilla et al. (Jiménez-Bonilla et al. 2021) deleted identified autolysin genes in *C. saccharoperbutyacetonicum*, observing characteristics similar to those shown here after the addition of phenolic acids. These included slightly increased solvent production, higher substrate consumption, and a slower progression compared to standard fermentation. Thus, delaying the intrinsic cell-suicide program enables cells to remain metabolically active for a longer period. Slower and prolonged solventogenesis may also help them to adapt to solvents by reducing the rate of their accumulation.

In *C. acetobutylicum*, genes related to sporulation, along with those encoding histidine kinases potentially involved in regulating sporulation initiation via Spo0A phosphorylation, were downregulated under phenolic stress (Luo et al. 2020, 2021). This downregulation may explain the reduced sporulation observed in all experiments involving phenolic challenges in this study. Conversely, a different ABE fermentation profile in the presence of furfural resulted in an increased sporulation rate and decreased solvent production, verifying that solventogenesis is not necessarily linked to sporulation in *C. beijerinckii* NRRL B598 (Branska et al. 2018). The regulation of sporulation in solventogenic clostridia remains an underexplored area, warranting further investigation, particularly regarding the influence of inhibitory substances (Diallo et al. 2021; Al-Hinai et al. 2015).

Unfortunately, exact regulatory mechanisms for most key processes have yet to be uncovered for solventogenic clostridia, despite extensive research. This gap in understanding may be attributed to the variability among producer strains, each possessing distinct genes, regulatory pathways, and genomic arrangements, even for critical processes such as energy acquisition and primary metabolite production (Poehlein et al. 2017). Variability is also apparent in how strains respond to inhibitors. The stimulatory effects of inhibitors vary across strains, with furan derivatives more frequently enhancing production. For instance, *C. beijerinckii* P260 demonstrated a significant improvement in production parameters when furfural and hydroxymethylfurfural (HMF) were added individually or in combination to the fermentation medium, up to concentrations of 1.5 g/L (Qureshi et al. 2012). Interestingly, higher production was paradoxically accompanied by reduced cell growth, a trend consistent with our observations for phenolic acids in *C. beijerinckii* NRRL B598. Conversely, a broader study including multiple strains reported growth promotion by furfural and HMF at concentrations ranging from 0.5 to 2.5 g/L (Ezeji and Blaschek 2008). Ezeji et al. (Ezeji et al. 2007) found that both furfural and HMF stimulated growth and production, with an approximate 19% improvement in *C. beijerinckii* BA101, while ferulic and coumaric acids had a distinctly negative effect on the strain. However, the concentration range tested in these studies was generally much higher than those used in our experiments. Among the five tested phenolic substances (including ferulic acid), only vanillin showed a stimulatory effect on the *C. acetobutylicum* ATCC 824 strain, increasing the final butanol titer by 10% (Luo et al. 2019).

One of the earlier studies on the impact of furfural on living organisms revealed its interaction with double-stranded DNA, causing single-strand breaks, primarily in AT­rich sequences (Hadi et al. 1989). This negative effect on DNA was indirectly confirmed through in vivo experiments with *C. beijerinckii*, where the organism’s ability to tolerate furfural improved significantly with the addition of allopurinol, which promotes purine salvage, potentially aiding DNA repair (Ujor et al. 2015). However, in this study, furfural was introduced to the growing culture at very high concentrations, starting at 4 g/L. Such concentrations are typically lethal to clostridia or prolong the lag phase from hours to several days (Zhang et al. 2012).

Based on our findings, we cannot confirm or exclude this effect. A clear decline in viability was observed, along with the effort of surviving cells to remove furfural. Cells that eventually recovered showed no significant changes in phenotype or production characteristics. Only after furfural was fully depleted from the medium, a steep increase in OD occurred. This aligns with previous observations, where the presence of furan derivatives significantly extended the lag phase. Following this delay, fermentation typically proceeded in a relatively standard manner, albeit with slightly altered yields and product concentrations (Zhang et al. 2012; Yao et al. 2017).

Furfural is transformed utilizing NAD(P)H dependent aldo/keto reductase (AKR) (Zhang et al. 2015; Okonkwo et al. 2019) and a short-chain dehydrogenase/reductase (SDR) to corresponding furfuryl alcohol, leading to redox balance perturbations (Zhang and Ezeji 2013; Ask et al. 2013; Ujor et al. 2014; Jilani and Olson 2023). Electrons used for furfural reduction may become deficient in key metabolic pathways (Ezeji et al. 2010), resulting in a redistribution of metabolic intermediates to other branches. This is clear from altered profile of butyric acid production in furfural challenged culture. Apparently, butyrate formation was severely suppressed, and the major contributor to the pH decline in this case was acetate, which peaked at the metabolic switch, resulting in a higher pH at the switch compared to the control. For both ferulic and coumaric acids, a similar mechanism is likely involved, where reduced cofactors like NADH or NADPH may facilitate their reduction in oxygen-independent biotransformations. Liu et al. (Liu et al. 2018) suggested that the Cbei_2996 gene (NADH-quinone oxidoreductase), which utilizes NADH, may play a crucial role in regulating p-coumaric acid tolerance in *C. beijerinckii* (Liu et al. 2018). Some *Clostridiales* members have demonstrated the capability to reduce ferulic and coumaric acids to their respective phenylpropionic acids (Chamkha et al. 2001). This is consistent with findings for *C. tyrobutyricum*, where dihydroferulic acid and phloretic acid were identified as biotransformation products (Luo et al. 2024). Phloretic acid has been identified as a reduced product of coumaric acid in cultures of *C. beijerinckii* too (Liu et al. 2018), suggesting its potential presence in our experiments as well. HLPC analysis revealed the formation of two new compounds with distinct absorption spectra and lower affinity for the hydrophobic stationary phase (data not shown). Both ferulic and coumaric acids were, however, rapidly converted, indicating that they were fully transformed well before the metabolic switch occurred.

Assuming a hypothetical transformation that consumes one mole of the reduced cofactor NADH per mole of phenolic acid (Filannino et al. 2014), and given the added phenolic acid concentration of 0.4 g/L, the corresponding amount of glucose required to supply sufficient electrons would be ≤ 0.22 g/L. Thus, it is unlikely that a shortage of reducing cofactors is the primary mechanism driving the observed changes in production behaviour. This is further supported by the unchanged acetate and butyrate production profiles during the early growth stages. The slower growth observed may instead be due to the energy required for stress response or the effects of biotransformation products present throughout the fermentation. Additionally, the presence of phenolic acids has often led to the acid-crash phenomenon. Only one such case is presented here; however, based on our extensive results, the susceptibility of cultures exposed to coumaric acid to acid-crash is high.

The interplay between the presence of acetic and butyric acids, reducing cofactors (NADH and NADPH) and the level of ATP are important for triggering solventogenesis (Wang et al. 2012, 2013; Zhao et al. 2005). Our observations clearly demonstrate that both acid concentrations and pH values can vary significantly depending on the conditions. The presence of phenolic substances causes a shift at higher acid concentrations and lower pH, while furfural induces a shift at lower acid concentrations and higher pH. Another notable distinction is the decrease in population viability observed after the addition of furfural, which was not observed with phenolic acids.

The hydrophobic nature of these substances is responsible for several negative effects, primarily affecting the membrane and its associated functions (Jiménez-Bonilla et al. 2024; Ibraheem and Ndimba 2013). Maintaining membrane integrity is crucial for cell survival, and this factor might play a key role in the loss of viability among populations more susceptible to furfural. Supporting the membrane associate pumps was shown to considerably alleviate the stress caused by lignocellulose inhibitors (Zou et al. 2022; Wan et al. 2023). Although furfural is considered the least toxic of all the tested substances (Yao et al. 2017), it was added at a higher concentration than the phenolic acids in this study, potentially amplifying its detrimental impact.

In addition to the aforementioned, the presence of inhibitors is associated with reduced expression of genes related to the PTS transport pathway, glycolytic and fermentative enzymes, altered regulation of genes involved in two-component signal transduction systems, and activation of general stress responses (Patakova et al. 2022; Liu et al. 2019, 2020; Suo et al. 2018). These factors collectively contribute to reduced cell replication rates and altered production characteristics. However, determining the precise causal relationships remains challenging.

Our findings indicate that the effects of furfural, coumaric acid, and ferulic acid on cell physiology, sporulation rates, and production parameters differ significantly. Interestingly, phenolic acids may also have a mildly stimulatory effect, likely due to the prolonged retention of cells in the production phase. In other words, the self-regulatory response of cells, which ultimately leads to self-sacrifice, was delayed. Further research is required to unravel the underlying mechanisms of this phenomenon and to develop strategies for achieving more substantial production improvements in the presence of these inhibitors.

## Electronic supplementary material

Below is the link to the electronic supplementary material.

Supplementary Material 1

## Data Availability

The data that support the findings of this study are included in this published article.
